# Pooled safety profiles of bispecific antibodies targeting PD-1/CTLA-4 or PD-1/VEGF in non-small cell lung cancer: a systematic review and single-arm meta-analysis

**DOI:** 10.1186/s43046-026-00387-2

**Published:** 2026-08-05

**Authors:** Jiayun Ma, Weixing Zhao

**Affiliations:** Graduate School of Qinghai University, Qinghai University, Xining, China

**Keywords:** Bispecific antibodies, Non-small cell lung cancer (NSCLC), PD-1/CTLA-4, PD-1/VEGF, Safety, Meta-analysis

## Abstract

**Objective:**

This study aimed to systematically synthesize and separately quantify the pooled safety profiles of bispecific antibodies (BsAbs) targeting PD-1/CTLA-4 or PD-1/VEGF in patients with non-small cell lung cancer (NSCLC), and to descriptively summarize class-specific safety patterns.

**Methods:**

Following PRISMA guidelines, we systematically reviewed prospective clinical trials published through January 2026. A total of 17 studies, encompassing 1802 patients, were included. Single-arm proportion meta-analyses were conducted separately for each BsAb class using random-effects models. No formal between-class statistical comparison was performed. Given the clinical heterogeneity across included cohorts, including differences in study phase, treatment line, molecular subtype, and treatment regimen, pooled estimates were interpreted descriptively. Subgroup analyses by treatment regimen, treatment line, and individual BsAb agent were considered exploratory. Because of the limited number of cohorts within each phase stratum, the impact of study phase was assessed descriptively rather than through formal stratified meta-analysis.

**Results:**

In separate pooled analyses, the incidence of grade 3 or higher treatment-related adverse events (TRAEs) was 41.79% for PD-1/CTLA-4 BsAbs and 43.51% for PD-1/VEGF BsAbs. Exploratory subgroup analyses suggested different patterns of heterogeneity across the available cohorts. In PD-1/CTLA-4 BsAb studies, AE rates appeared to vary across individual agents, whereas in PD-1/VEGF BsAb studies, higher AE rates were observed mainly in chemotherapy-containing regimens. These observations should be interpreted cautiously because they may reflect differences in the distribution of included agents, patient populations, study phases, and treatment strategies rather than inherent class-specific toxicity mechanisms. Regarding tolerability, the pooled treatment discontinuation rate due to TRAEs was 10.26% in the PD-1/CTLA-4 analysis and 4.44% in the PD-1/VEGF analysis. The pooled incidence of immune-related adverse events (irAEs) was 46.68% in the PD-1/CTLA-4 analysis and 27.10% in the PD-1/VEGF analysis, whereas the pooled rates of grade 3 or higher irAEs were low in both analyses.

**Conclusion:**

This systematic review provides a descriptive synthesis of available safety data for PD-1/CTLA-4 and PD-1/VEGF BsAbs in NSCLC. Because the included cohorts differed substantially in study phase, treatment line, molecular background, and concomitant therapies, the pooled AE estimates should not be interpreted as direct comparative evidence. Instead, the findings should be viewed as hypothesis-generating signals that require validation in more homogeneous prospective studies or head-to-head trials.

**Supplementary Information:**

The online version contains supplementary material available at 10.1186/s43046-026-00387-2.

## Introduction

 Lung cancer remains the leading cause of cancer-related morbidity and mortality worldwide, and non-small cell lung cancer (NSCLC) accounts for approximately 85% of all cases [1, 2]. The therapeutic landscape of NSCLC has been revolutionized over the past decade, propelled largely by the emergence of immunotherapy [3]. A pivotal breakthrough in this field involved targeting immune checkpoints, which are crucial immunomodulatory pathways for maintaining immune homeostasis and regulating the body’s immune response [4]. Consequently, the clinical integration of immune checkpoint inhibitors (ICIs), particularly those targeting programmed cell death protein 1 (PD-1) and its ligand (PD-L1), has markedly improved outcomes for patients with advanced, driver-gene-negative NSCLC. Landmark clinical trials, such as KEYNOTE-189 and KEYNOTE-407, have established immunotherapy, either as monotherapy or combined with chemotherapy, as a first-line standard of care, delivering significant extensions in overall survival [5, 6]. Despite these advances, formidable challenges remain. A durable objective response to ICI monotherapy is achieved in only 20–30% of unselected patients, and the majority ultimately experience disease progression due to primary or acquired resistance [7]. Furthermore, the efficacy of immunotherapy is notably limited in specific clinical subgroups, including patients with low PD-L1 expression or a “cold tumor” phenotype. This context highlights the urgent need for novel combination strategies capable of remodeling the tumor immune microenvironment to broaden the therapeutic benefit of immunotherapy.

To overcome the efficacy limitations of ICI monotherapy, dual blockade of non-redundant immunosuppressive pathways represents a promising therapeutic avenue. One strategy combines PD-1 and CTLA-4 inhibition (e.g., nivolumab plus ipilimumab), which provides mechanistic complementarity by targeting the distinct priming and effector phases of the T-cell response [8]. Another approach pairs anti-angiogenic agents targeting VEGF or its receptor (VEGFR) with ICIs. Blockade of the VEGF pathway demonstrates synergistic effects; it not only normalizes tumor vasculature but also mitigates the function of regulatory T-cells (Tregs) and myeloid-derived suppressor cells (MDSCs), thereby helping to remodel the immunosuppressive tumor microenvironment [9]. Although these dual-agent regimens improve efficacy, they also introduce significant safety concerns. Dual ICI blockade, for instance, is associated with a higher incidence of grade ≥ 3 irAEs, which can limit its use in vulnerable populations, such as the elderly or patients with poor performance status [10]. Therefore, a central challenge in developing next-generation immunotherapies is to enhance anti-tumor activity while concurrently mitigating systemic toxicity.

The emergence of bispecific antibodies (BsAbs) offers an innovative solution to the challenge of balancing efficacy with toxicity. Unlike the co-administration of two separate monoclonal antibodies, a single BsAb is engineered to engage two distinct targets concurrently. The two BsAb classes at the forefront of clinical development in NSCLC target either PD-1/CTLA-4 (e.g., Cadonilimab, KN046) or PD-1/VEGF (e.g., Ivonescimab/AK112). These agents are designed to leverage a “cis-binding” mechanism, which facilitates preferential binding to cells that co-express both targets, such as tumor cells and tumor-infiltrating lymphocytes. This approach aims to enhance intratumoral drug concentration while mitigating off-target effects, thereby widening the therapeutic window [11, 12]. Recent pivotal clinical trials have begun to validate this design philosophy. For instance, data from the phase III HARMONi-2 study demonstrated that the PD-1/VEGF BsAb, Ivonescimab, achieved superiority over pembrolizumab in patients with PD-L1-positive NSCLC. This finding represents a significant milestone for the BsAb field and advances the exploration of “chemotherapy-free” regimens [13]. Concurrently, PD-1/CTLA-4 BsAbs have shown promising activity as a salvage therapy in patients with treatment-refractory NSCLC [14]. 

However, the increasing clinical adoption of BsAbs has also underscored the complexity of their safety profiles. The divergent mechanisms of PD-1/VEGF and PD-1/CTLA-4 BsAbs may give rise to different adverse event (AE) spectra. Specifically, PD-1/VEGF inhibition is associated with angiogenesis-related toxicities (e.g., hypertension, proteinuria), while dual immune checkpoint blockade with PD-1/CTLA-4 BsAbs necessitates vigilance for a broader range of irAEs [15]. Although individual trials have reported safety data, the overall safety burden of PD-1/CTLA-4 and PD-1/VEGF BsAbs has not been systematically synthesized in the NSCLC population. Therefore, we conducted a systematic review and single-arm proportion meta-analysis to separately synthesize the available safety data for PD-1/CTLA-4 and PD-1/VEGF BsAbs in NSCLC. We also explored potential sources of heterogeneity, including study phase, individual BsAb agent, treatment line, molecular subtype, and treatment regimen. Given the indirect nature of these observations and the heterogeneity of included cohorts, our aim was not to formally compare AE incidences across disparate cohorts or to define biological distinctions between BsAb classes, but to provide a descriptive and hypothesis-generating summary of current safety evidence.

## Materials and methods

### Search strategy and study selection

This systematic review and meta-analysis was designed and conducted in accordance with the Preferred Reporting Items for Systematic Reviews and Meta-Analyses (PRISMA) guidelines [16]. The study protocol was registered with the International Prospective Register of Systematic Reviews (PROSPERO; registration number: CRD420261293100).We performed a systematic search of the PubMed, EMBASE, Cochrane Library, and Web of Science databases for relevant studies published from inception to January 10, 2026, with the search restricted to articles in English. The search was supplemented by a manual review of reference lists from retrieved articles and abstracts from major oncology conferences, including the American Society of Clinical Oncology (ASCO), the World Conference on Lung Cancer (WCLC), the European Society for Medical Oncology (ESMO), and the American Association for Cancer Research (AACR).The search strategy combined terms for the disease (“non-small cell lung cancer”), intervention (“bispecific antibody,” “PD-1,” “PD-L1,” “CTLA-4,” “VEGF”), and specific drug names (e.g., cadonilimab, KN046, ivonescimab, PM8002). Retrieved records were managed using EndNote X9. Two authors independently screened titles, abstracts, and full texts for eligibility. Any discrepancies were resolved through discussion or, if necessary, adjudication by a third author to reach a consensus.

### Inclusion and exclusion criteria

Studies were included if they met the following criteria: (1) Population: Patients diagnosed with NSCLC; (2) Intervention: Treatment with a BsAb targeting either PD-1/CTLA-4 (e.g., cadonilimab, KN046) or PD-1/VEGF (e.g., ivonescimab, PM8002), administered as monotherapy or in combination with other agents; (3) Study Design: Prospective clinical trials, including full-text articles and conference abstracts; and (4) Outcomes: Reported data on at least one safety endpoint, such as TRAEs, grade 3 or higher TRAEs, treatment discontinuation due to TRAEs, serious adverse events (SAEs), or irAEs. Only studies published in English were considered. We excluded studies that enrolled mixed tumor cohorts where NSCLC data could not be disaggregated. Additionally, non-prospective study designs, preclinical research, and reports lacking extractable safety data were excluded. For duplicate publications originating from the same clinical trial, the report with the most recent or comprehensive data was retained for analysis. Because the current clinical evidence for BsAbs in NSCLC remains limited and is derived from diverse clinical settings, we included both monotherapy and combination-regimen cohorts to comprehensively capture available safety data. However, these cohorts were not assumed to be directly comparable. Therefore, AE incidences from monotherapy cohorts, chemotherapy-containing cohorts, EGFR-mutated cohorts, and other combination-regimen cohorts were interpreted descriptively and in the context of the study-level information available from the published reports.

### Data extraction

For each included study, we extracted the study name, author, year of publication, BsAb class, incidence rates of adverse events, reported safety endpoints, and other details required for the planned single-arm proportion meta-analysis. To contextualize clinical heterogeneity, we also recorded readily available study-level descriptors as reported in the eligible publications and summarized in Table 1, including study phase, individual BsAb agent, sample size, treatment line, treatment regimen, and molecular subgroup or EGFR mutation status when explicitly reported. Information on chemotherapy exposure and other combination agents was used descriptively when it was identifiable from the published regimen description. Unreported variables were not imputed, and these descriptors were not used to construct a new formal comparative dataset across heterogeneous cohorts. Data extraction was performed independently by two authors. Any discrepancies were resolved through discussion with a third author until a consensus was reached.

**Table 1:** Characteristics of studies included in this network meta-analysis

Study	Year	Auther	Phase	Intervention	TEAE %	≥ 3TEAE %	TRAE %	≥ 3TRAE %	Serious TRAE %	Discontinuation TRAE	irAE %	≥ 3 irAE %
NCT05142423	2025	Y. Zheng	Ib/II	Cadonilimab+Pulocimab 47	-	36.2	93.6	21.3	23.4	4.3	19.1	6.4
NCT05329025	2025	Y. Zhang	II	QL1706་CT་Bev 31	-	-	96.8	41.9	35.5	6.5	61.3	3.2
NCT06424821/ LungCadX	2024	L. Wang	II	Cadonilimab + CT 44	68.2	34.1	61.4	34.1	13.6	2.3	18.2	-
NCT05420220	2024	Y. Zhao	II	KN046 + Axitinib 85	98.8	67.1	96.5	58.8	45.9	-	24.7	10.6
NCT03530397	2024	D.R. Spigel	Ib	Volrustomig་CT 139	-	-	97.1	75.7	-	-	-	-
NCT05329025	2024	Y. Huang	II	QL1706་CT 17	-	-	100	17.6	23.5	-	82.4	5.9
				QL1706་CT 11	-	-	100	36.4%	18.2%	9.1%	63.6	-
				QL1706་CT 12	-	-	100	16.7	16.7	-	66.7	8.3
				QL1706་CT་Bev 20	-	-	90.0	50.0	35.0	20.0	65.0	5.0
NCT04054531	2024	Y. Zhao	II	KN046 + CT 87	100	72.4	100	66.7	31.0	19.5	57.5	12.6
NCT03838848	2023	A. Xiong	II	KN046 64	-	-	92.2	42.2	32.8	14.1	60.9	20.3
NCT03838848			II	KN046 + CT 26	-	-	92.3%	57.7%	-	-	-	-
NCT04646330	2023	B. Chen	Ib/II	Cadonilimab+anlotinib 69	-	-	97.1	49.3	50.7	13.0	20.3	5.8
NCT04172454	2023	Y. Zhao	Ib/II	Cadonilimab53	92.5	24.5	73.6	11.3	-	-	-	1.9
NCT05184712/ HARMONi-A	2025	L. Zhang	Ⅲ	ivonescimab + CT 161	99.4	67.1	98.8	59.6	32.3	11.2	24.2	6.2
NCT05499390/ HARMONi-2	2025	A. Xiong	Ⅲ	Ivonescimab 197	-	-	90	29	21	2	30	7
NCT06396065/HARMONi(Global)	2025	J.W. Goldman	Ⅲ	ivonescimab + CT 218	-	-	95	50	28	7.3	33	9.6
HARMONi-6	2025	Z. Chen	Ⅲ	Ivonescimab + CT 266	-	-	99.2	63.9	32.3	3.4	27.4	9.0
NCT04900363/ HARMONi-5	2024	L. Wang	Ib	Ivonescimab 108	98.1	38.0	91.7	22.2	22.2	0.9	22.2	3.7
NCT05756972	2024	Y. Wu	II	PM8002 + CT 64	-	-	95.3	54.7	-	9.4	28.1	4.7
NCT04736823/ AK112-201	2023	Y. Zhao	II	ivonescimab + CT 83	100.0	51.8	91.6	26.5	-	2.4	16.9	2.4

### Quality and risk of bias assessment

The methodological quality and risk of bias of all included studies were independently assessed by two authors, with any discrepancies resolved through discussion to reach a consensus.For randomized controlled trials (RCTs), we utilized the Cochrane Risk of Bias tool [17]. This instrument evaluates potential bias across seven domains: random sequence generation, allocation concealment, blinding of participants and personnel, blinding of outcome assessors, incomplete outcome data, selective reporting, and other sources of bias. Each domain was judged as having a “low,” “high,” or “unclear” risk of bias, and the results were visualized in a summary figure. For non-randomized studies of interventions (NRSIs), such as single-arm trials, quality was assessed using the Newcastle-Ottawa Scale (NOS) [18]. 

### Statistical analysis

Independent single-group proportion meta-analyses were conducted using the ‘meta’ package in R (version 4.5.1). Pooled estimates and their corresponding 95% confidence intervals (CIs) were calculated. Proportion data were transformed using the Freeman-Tukey double arcsine method, and the results were displayed using forest plots. Statistical heterogeneity among studies was quantified using the I2 statistic. Because the included evidence consisted largely of independent single-arm cohorts and no randomized head-to-head trials directly comparing PD-1/CTLA-4 with PD-1/VEGF BsAbs were available, we did not conduct a formal between-class comparative meta-analysis. Therefore, differences observed between pooled estimates for the two BsAb classes were interpreted descriptively and should be considered hypothesis-generating rather than confirmatory. Clinical heterogeneity was anticipated because the included cohorts differed in study phase, treatment line, molecular background, and treatment regimen, including EGFR-mutated populations and chemotherapy-containing combinations. Therefore, random-effects models were used for all pooled estimates. To explore potential sources of heterogeneity, subgroup analyses were performed according to treatment regimen, treatment line, and individual BsAb agent where data were available. EGFR mutation status, chemotherapy-containing regimens, and study phase were also considered in descriptive interpretation. Because the number of cohorts within several phase-specific strata was limited, formal stratified meta-analysis by study phase was not feasible for all endpoints. Publication bias and small-study effects were assessed through visual inspection of funnel plots and formally evaluated using Begg’s and Egger’s tests. A statistically significant Egger’s test was interpreted as evidence of funnel plot asymmetry or possible small-study effects rather than definitive proof of publication bias. Publication-bias assessments were interpreted together with heterogeneity statistics, subgroup findings, and sensitivity analyses. A p-value of less than 0.05 was considered statistically significant.

## Results

### Literature search and study selection

**Fig. 1:**
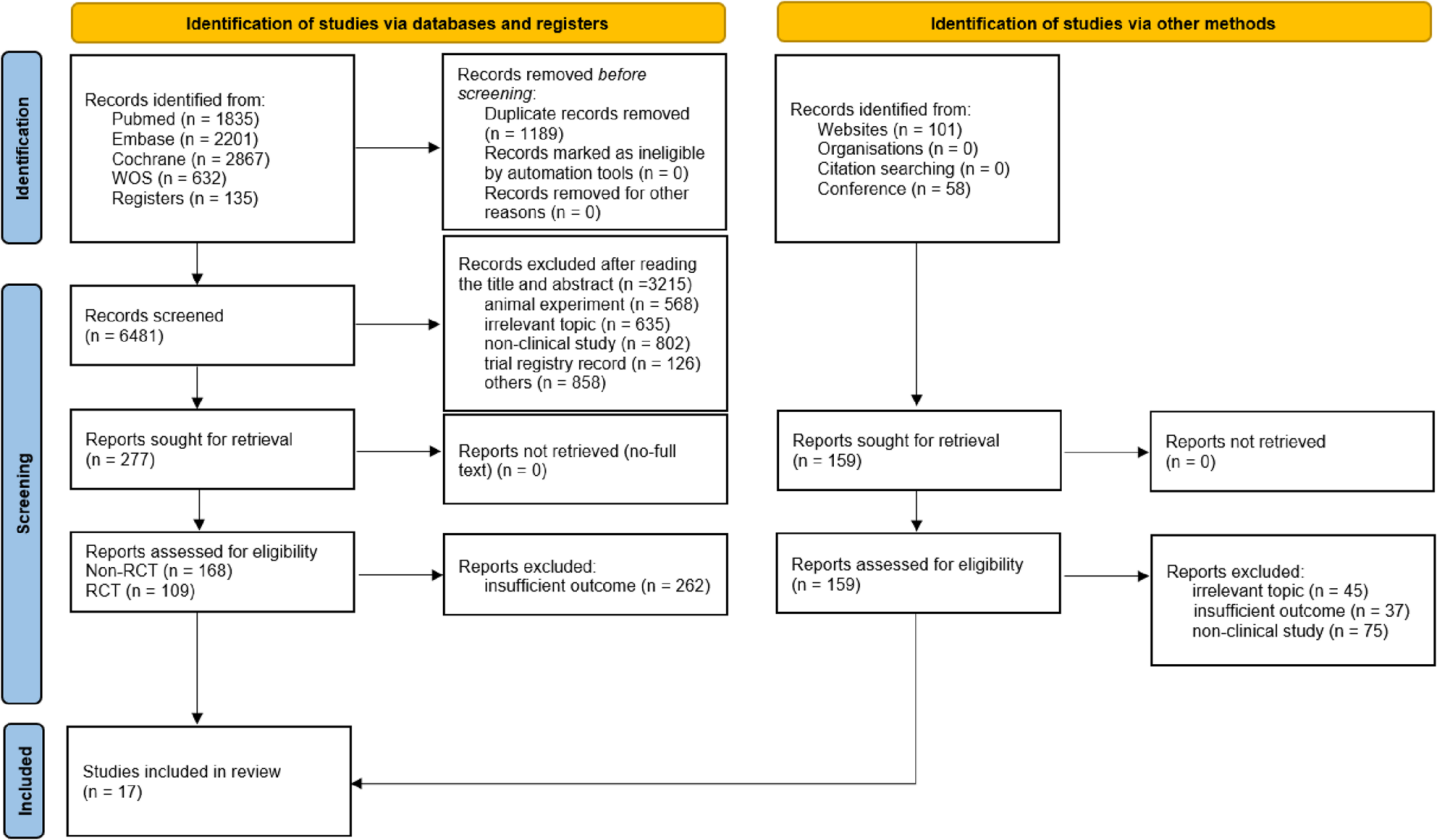
Flow diagram of the meta-analysis for inclusion and exclusion of studies. Randomized controlled trials (RCTs)

The initial database search yielded 7,829 records. After the removal of 1,189 duplicates, 6,640 unique records were screened based on their titles and abstracts. During this screening stage, 6,363 records were excluded for not meeting the inclusion criteria (e.g., being preclinical studies, reviews, or irrelevant to the topic). This process left 277 articles for a detailed full-text review.Following full-text assessment, 260 articles were further excluded based on the predefined eligibility criteria. Ultimately, a total of 17 studies, comprising four randomized controlled trials (RCTs) [13, 19–21] and 13 non-randomized, single-arm studies [14, 22–33], met the criteria for inclusion in the qualitative synthesis and meta-analysis. The complete study selection process is illustrated in the PRISMA flowchart in Fig. 1.

### Study characteristics

Seventeen prospective clinical studies were included in the meta-analysis, comprising four RCTs and 13 single-arm cohorts that involved 1802 patients diagnosed with non-small cell lung cancer. These clinical trials included two Phase Ib [26, 31], three Phase Ib/II [22, 29, 30], eight Phase II [14, 23–25, 27, 28, 32, 33] and four Phase III studies [13, 19–21]. The included studies were published between 2023 and 2025, and the specific characteristics of each study are presented in Table 1.

### Quality assessment

The methodological quality of the included studies was rigorously evaluated. For the four RCTs, the Cochrane Risk of Bias tool indicated an overall low risk of bias (Supplementary Fig. 1). A potential for performance and detection bias was noted across all RCTs, an unavoidable consequence of their open-label design, which precludes the blinding of participants and personnel. Several domains were judged as having an “unclear” risk, primarily stemming from insufficient reporting on specific methodological details. However, key areas such as random sequence generation and attrition bias were consistently rated as low risk.The quality of the 13 non-randomized studies was assessed using the Newcastle-Ottawa Scale (NOS). The majority of these trials were determined to be of high quality, scoring well in the domains of cohort selection and outcome ascertainment. The detailed NOS scores for each study are available in Supplementary Table 1.

## Meta-analysis results

### TRAEs

#### Incidence of all-grade TRAEs

Our analysis of all-grade TRAEs incorporated data from 14 study cohorts evaluating PD-1/CTLA-4 BsAbs and seven cohorts evaluating PD-1/VEGF BsAbs. The meta-analysis demonstrated a substantial pooled incidence of TRAEs in both independent evidence groups. Because the included studies differed in study phase, treatment line, EGFR mutation status, and concomitant systemic therapy, numerical differences across BsAb classes were interpreted cautiously and were not treated as direct comparative effect estimates.

**Fig. 2:**
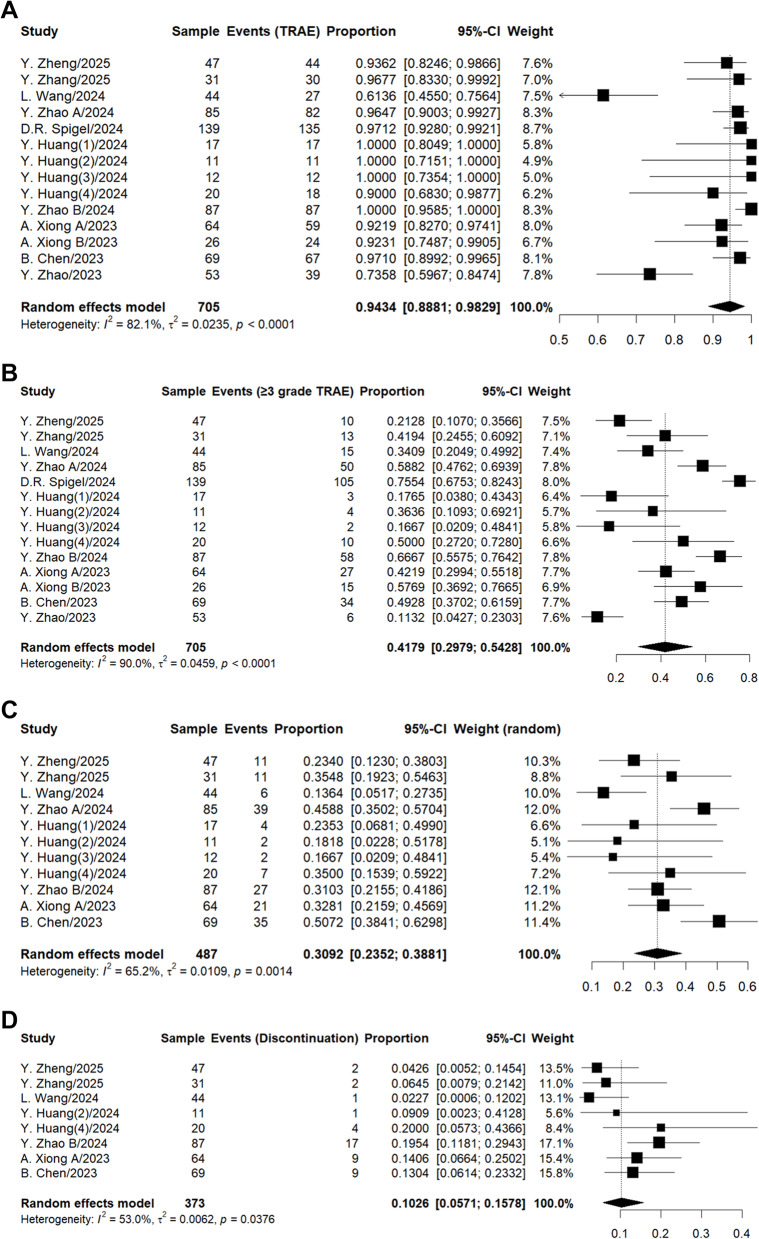
Forest plots of PD-1/CTLA-4 BsAb-related TRAEs. **A** Overall TRAE. **B** ≥3grade TRAE. **C** Serious TRAE. **D** Discontinuation TRAE

For PD-1/CTLA-4 BsAbs, the random-effects model revealed a substantial burden of all-grade TRAEs, accompanied by high between-study heterogeneity (I2 = 82.1%, *p* < 0.0001; Fig. 2). Exploratory subgroup analyses suggested that the specific BsAb agent may contribute to this heterogeneity (Figures S8, S11, S14). TRAE rates were higher in cohorts treated with QL1706 or KN046 than in those treated with Cadonilimab. This pattern may be compatible with agent-related safety variation, but it should not be viewed as definitive because cohorts also varied by study phase, dose level, treatment regimen, treatment line, sample size, and patient selection.

**Fig. 3:**
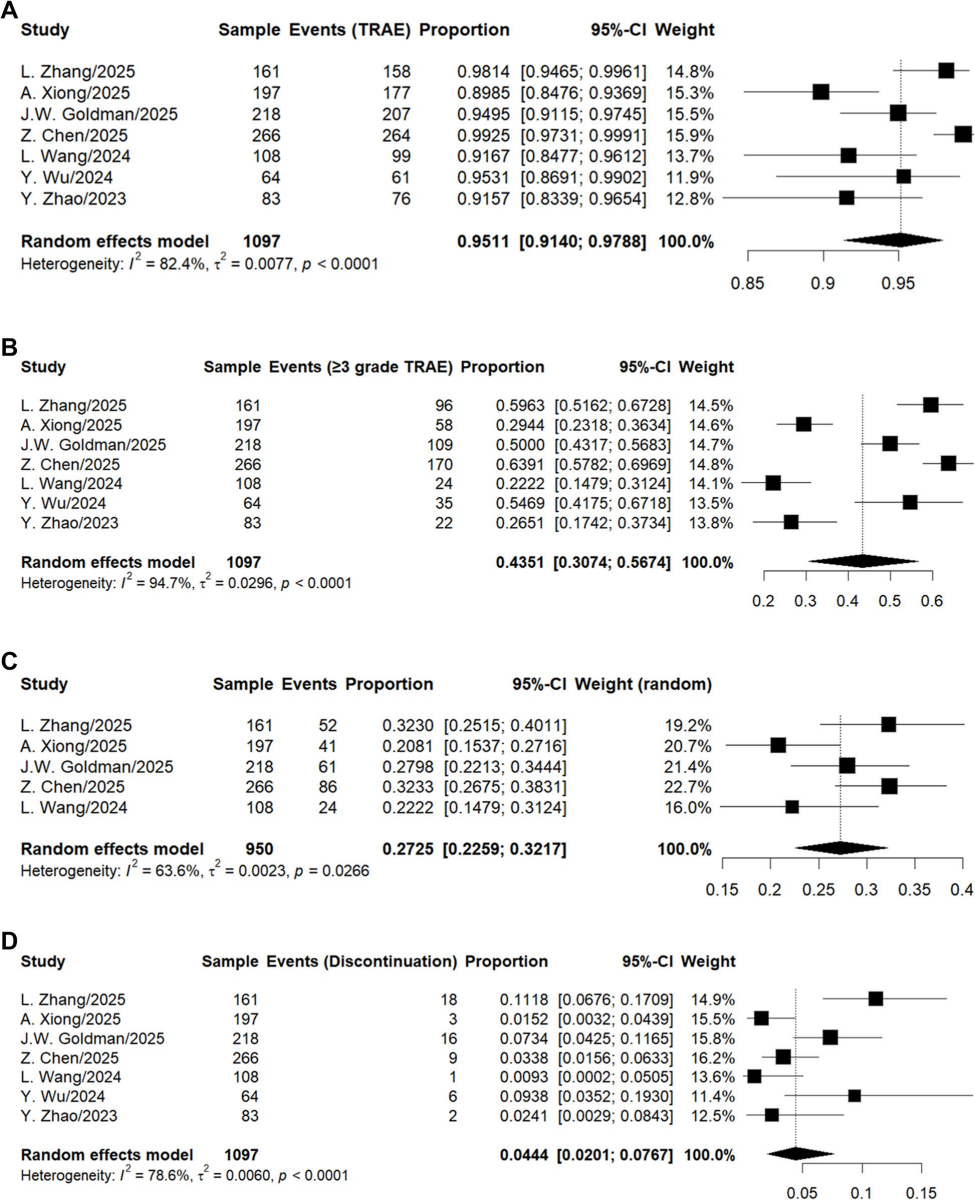
Forest plots of PD-1/VEGF BsAb-related TRAEs. **A** Overall TRAE. **B** ≥3grade TRAE. **C** Serious TRAE. **D** Discontinuation TRAE

For PD-1/VEGF BsAbs, the pooled incidence of all-grade TRAEs was 95.11% (95% CI: 91.40%-97.88%), with similarly high heterogeneity (I2 = 82.4%, *p* < 0.0001; Fig. 3). Within this evidence group, TRAE incidence appeared higher in chemotherapy-containing regimens than in monotherapy cohorts, whereas no clear difference was observed between individual agents (i.e., Ivonescimab vs. PM8002). Taken together, the separate subgroup analyses suggested different apparent patterns of heterogeneity across the included cohorts. However, this interpretation should be considered exploratory because these patterns may reflect differences in the distribution of included agents, treatment regimens, study populations, study phases, and treatment lines rather than a fixed class-specific toxicity biology (Figures S9, S12, S15). Study phase may also have contributed to the substantial heterogeneity observed for all-grade TRAEs, because early-phase studies may report higher or more variable all-grade AE rates because of dose-escalation designs, broader dose ranges, smaller sample sizes, and intensive safety monitoring.

#### Profile of severe toxicity

Separate pooled analyses of severe AEs, including grade 3 or higher TRAEs and SAEs, were conducted for each BsAb class. Because the included cohorts differed in treatment line, EGFR mutation status, study phase, and use of concomitant chemotherapy, the pooled estimates were interpreted descriptively rather than as directly comparable severe-toxicity rates. For PD-1/CTLA-4 BsAbs, the pooled incidence of grade 3 or higher TRAEs was 41.79% (95% CI: 29.79%-54.28%; Fig. 2), with significant between-study heterogeneity (*p* < 0.0001). Exploratory subgroup analyses suggested agent-specific variation, with Volrustomig showing the highest incidence. The pooled incidence of SAEs was 30.92% (Fig. 2). This observation suggests that individual BsAb-related factors may contribute to variability in severe toxicity among PD-1/CTLA-4 BsAb studies. However, given differences in study phase, dose level, treatment line, patient population, and concomitant therapy, the pooled estimates should not be interpreted as reflecting only the toxicity of dual immune-checkpoint blockade itself. For PD-1/VEGF BsAbs, the pooled incidence of grade 3 or higher TRAEs was 43.51% (95% CI: 30.74%-56.74%; Fig. 3), and the incidence of SAEs was 27.25% (Fig. 3). Subgroup analysis suggested that treatment regimen may be an important contributor to severe toxicity in PD-1/VEGF BsAb studies. Higher pooled rates were observed in chemotherapy-containing cohorts, but this pattern may also be confounded by differences in treatment line, EGFR mutation status, prior treatment exposure, baseline patient risk, and study design (Figures S9, S12, S15). Overall, the subgroup findings suggest that severe-toxicity heterogeneity may be associated with different factors across the available cohorts, including individual BsAb agents, treatment regimens, and study phase. These findings should be interpreted as exploratory observations rather than evidence that PD-1/CTLA-4 toxicity is inherently agent-dependent or that PD-1/VEGF toxicity is inherently regimen-dependent.

#### Treatment tolerability and discontinuation

The rate of treatment discontinuation due to TRAEs is a key indicator of clinical tolerability, but it is influenced not only by drug toxicity but also by treatment setting, protocol-defined discontinuation rules, follow-up duration, and investigators’ management strategies. For PD-1/CTLA-4 BsAbs, the pooled discontinuation rate was 10.26% (95% CI: 5.71%-15.78%; Fig. 2). Subgroup analysis suggested possible drug-specific differences, with higher discontinuation in KN046 cohorts and lower discontinuation in Cadonilimab cohorts (Figures S8, S11, S14). In the separate PD-1/VEGF analysis, the pooled discontinuation rate due to TRAEs was 4.44% (95% CI: 2.01%-7.67%; Fig. 3). This numerically low rate was mainly observed in available PD-1/VEGF cohorts, but should not be interpreted as evidence of a between-class tolerability advantage because the included cohorts differed in treatment regimen, treatment line, molecular background, study phase, and concomitant therapy. This finding suggests that PD-1/VEGF BsAb monotherapy cohorts may have a favorable discontinuation profile in the currently available evidence. Nevertheless, this observation requires confirmation in prospective studies with homogeneous populations and standardized treatment regimens.

#### Publication bias and sensitivity analysis

The potential for publication bias and small-study effects was evaluated across TRAE-related endpoints. Visual inspection of funnel plots showed generally symmetrical distributions for most analyses (Figures S2-S3). Formal testing was also non-significant for most endpoints. However, Egger’s test was statistically significant for grade 3 or higher TRAEs in the PD-1/CTLA-4 analysis (*p* = 0.024), suggesting possible funnel plot asymmetry. This finding indicates that publication bias or small-study effects cannot be excluded for this endpoint. Nevertheless, funnel plot asymmetry should not be interpreted as definitive evidence of publication bias alone, particularly in the presence of substantial clinical and statistical heterogeneity. The PD-1/CTLA-4 grade 3 or higher TRAE analysis included cohorts that differed in individual BsAb agent, study phase, dose level, treatment line, concomitant therapy, follow-up duration, and sample size. Therefore, the observed asymmetry may reflect small-study effects or genuine between-study heterogeneity rather than selective publication alone. Leave-one-out sensitivity analyses suggested that the pooled estimate was not driven by any single study; however, the significant Egger’s test supports cautious interpretation of the pooled incidence estimate for grade 3 or higher TRAEs in the PD-1/CTLA-4 subgroup.

### irAEs

#### Overall incidence of irAEs

The analysis of irAEs included 11 cohorts treated with PD-1/CTLA-4 BsAbs and seven cohorts treated with PD-1/VEGF BsAbs. Separate pooled analyses suggested different numerical patterns of irAE incidence across the available PD-1/CTLA-4 and PD-1/VEGF cohorts. However, because the included populations, study phases, and treatment regimens were heterogeneous, these estimates should not be interpreted as direct evidence of between-class differences in immunotoxicity.

**Fig. 4:**
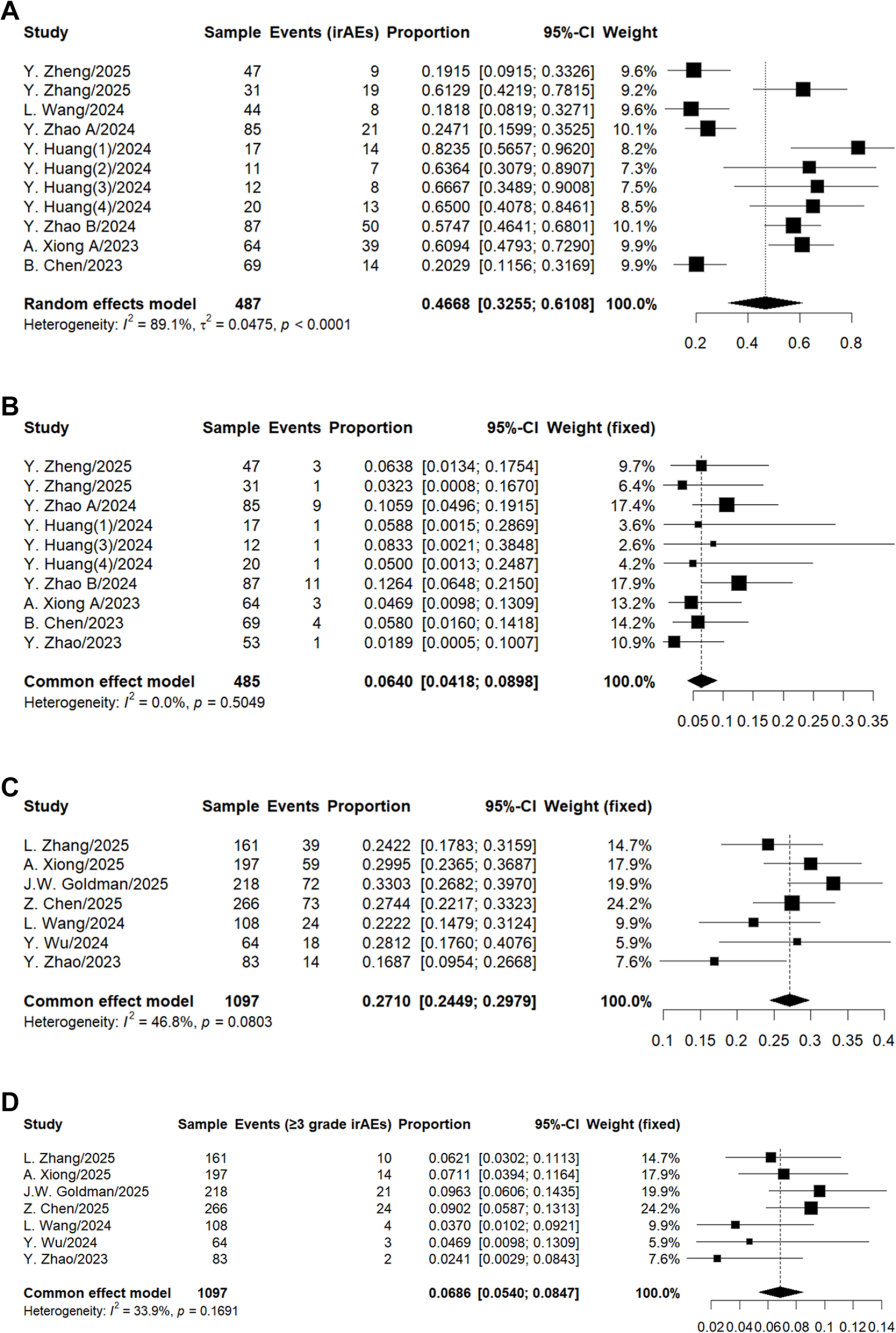
Forest plots of irAEs. **A** PD-1/CTLA-4 Overall irAE. **B** PD-1/CTLA-4 ≥3grade irAE. **C** PD-1/VEGF Overall irAE. **D** PD-1/VEGF ≥3grade irAE

For PD-1/CTLA-4 BsAbs, the random-effects model yielded a pooled irAE incidence of 46.68% (95% CI: 32.55%-61.08%), with substantial between-study heterogeneity (I2 = 89.1%, *p* < 0.0001; Fig. 4). Exploratory subgroup analyses suggested that heterogeneity may be related to individual BsAb agents, whereas no consistent modulation by treatment regimen or line of therapy was observed (Figures S10, S13, S16). This finding suggests that individual BsAb-related factors may contribute to variability in irAE incidence among PD-1/CTLA-4 BsAb studies. However, the high heterogeneity indicates that other factors, including study phase, treatment setting, AE ascertainment, prior therapy exposure, combination therapy, and follow-up duration, may also have influenced the reported irAE rates. For PD-1/VEGF BsAbs, the pooled irAE incidence was 27.10% (95% CI: 24.49%-29.79%; Fig. 4), with lower heterogeneity (I2 = 46.8%, *p* = 0.0803). This pooled estimate was numerically lower than the estimate observed in the separate PD-1/CTLA-4 analysis. However, no formal between-class comparison was performed, and differences in treatment setting and concomitant therapies may have influenced the observed rates. Because irAE definitions, monitoring intensity, and reporting practices may differ across trials, the pooled incidence of overall irAEs should be interpreted cautiously, particularly for low-grade irAEs.

#### Grade ≥ 3 irAEs

In the separate pooled analyses, grade 3 or higher irAEs occurred at low pooled rates in both BsAb classes. In contrast to the high heterogeneity observed for overall irAEs, heterogeneity was low to moderate for grade 3 or higher irAEs, which may reflect more consistent recognition, attribution, and reporting of severe events across trials. Therefore, the pooled estimates for grade 3 or higher irAEs may be more clinically interpretable than those for all-grade irAEs, although they remain limited by the small number of available cohorts.

The pooled incidence of grade 3 or higher irAEs for PD-1/CTLA-4 BsAbs was 6.40% (95% CI: 4.18%-8.98%, I2 = 0.0%) (Fig. 4). Although differences between individual drugs did not reach statistical significance, the treatment-regimen subgroup showed a possible signal: severe irAE risk was higher in combination-therapy cohorts than in monotherapy cohorts, approaching the threshold for statistical significance (*p* = 0.0513). This observation should be interpreted cautiously because combination-therapy cohorts may differ from monotherapy cohorts in patient population, prior therapy exposure, and treatment setting. The pooled incidence of grade 3 or higher irAEs for PD-1/VEGF BsAbs was 6.86% (95% CI: 5.40%-8.47%, I2 = 33.9%) (Fig. 4). No statistically significant subgroup differences were detected by individual drug, treatment regimen, or line of therapy, but sparse event counts limit the certainty of these subgroup findings.

Taken together, the separate analyses suggest that PD-1/CTLA-4 BsAbs may be associated with more frequent low-grade immune reactions, whereas grade 3 or higher irAE rates remained low in the available cohorts. The available PD-1/VEGF BsAb cohorts showed relatively consistent pooled estimates for irAEs. These observations remain descriptive because the studies differed in study phase, patient population, treatment setting, safety reporting, and concomitant therapies.

#### Publication bias and sensitivity analysis

The assessment of publication bias for irAE-related endpoints showed no significant asymmetry by Egger’s test (Supplementary Table 3). However, Begg’s test was significant for grade 3 or higher irAEs in the PD-1/VEGF subgroup (*p* = 0.038), while Egger’s test for the same endpoint was not significant (*p* = 0.110). This isolated signal may reflect small-study effects, sparse events, or heterogeneity. Therefore, funnel-plot based assessments should be interpreted cautiously. The sensitivity analysis confirmed that the pooled effect estimates were not materially altered by the removal of any single study (Figure S7).

### Descriptive assessment of study phase

Formal sensitivity analysis stratified by study phase was limited by the small number of cohorts within several phase-specific strata and by incomplete reporting of endpoints across phases. Therefore, we assessed the potential impact of study phase descriptively. Early-phase studies may contribute disproportionately to heterogeneity because they often include smaller sample sizes, broader dose ranges, dose-escalation or dose-expansion designs, and more intensive AE monitoring. In contrast, phase III trials generally include larger and more selected populations treated at recommended doses under standardized protocols. Thus, pooled estimates that combine early-phase and late-phase studies should be interpreted as descriptive summaries rather than phase-specific safety estimates.

## Discussion

To our knowledge, this is the first systematic review and single-arm proportion meta-analysis to separately synthesize the safety profiles of PD-1/CTLA-4 and PD-1/VEGF BsAbs in NSCLC. A central issue in interpreting our findings is the substantial heterogeneity observed in several pooled analyses, particularly all-grade TRAEs and overall irAEs. This heterogeneity is clinically expected given the diversity of the included evidence, which spans early-phase and phase III trials, different treatment lines, molecularly selected populations, EGFR-mutated post-TKI cohorts, treatment-naive cohorts, BsAb monotherapy, and chemotherapy-containing combinations. Therefore, the pooled incidence estimates should be interpreted as descriptive summaries of the current evidence rather than precise safety rates generalizable to all patients or treatment settings. Within this limitation, subgroup analyses suggested that heterogeneity may be influenced by individual BsAb agents, treatment regimen, study phase, and patient population; however, these findings remain exploratory and hypothesis-generating.

Several factors may explain the high heterogeneity observed in this meta-analysis. First, study phase may have contributed to variability. Early-phase studies often include dose-escalation or dose-expansion cohorts, smaller sample sizes, broader eligibility criteria, and more intensive safety monitoring, whereas phase III trials generally enroll larger and more selected populations under standardized protocols. Second, patient populations differed substantially. Some studies enrolled treatment-naive patients, whereas others included previously treated patients or EGFR-mutated patients after EGFR-TKI failure. These populations may have different baseline organ function, prior toxicity exposure, immune microenvironment, and vulnerability to treatment-related AEs. Third, treatment settings varied across trials. BsAb monotherapy, chemotherapy-containing combinations, anti-angiogenic combinations, and other targeted combinations are unlikely to have identical AE profiles. Chemotherapy, in particular, can independently increase hematologic, gastrointestinal, and constitutional toxicities, thereby increasing the pooled safety burden. Fourth, individual BsAb agents differ in molecular design, including target affinity, valency, Fc-domain engineering, and binding configuration, which may influence both immune activation and off-target toxicity. Finally, differences in follow-up duration, AE definitions, attribution rules, and reporting intensity may have contributed to variability, especially for all-grade AEs and low-grade irAEs. Accordingly, pooled incidence estimates in this study should not be interpreted as fixed toxicity rates for a given BsAb class. Rather, they provide an overview of the safety burden reported in currently available trials and help identify areas where heterogeneity should be addressed in future studies.

The clinical utility of conventional CTLA-4 inhibitors such as ipilimumab has been limited by severe irAEs, including colitis and hypophysitis, which have been linked to systemic immune activation and regulatory T-cell depletion [34, 35]. In the available PD-1/CTLA-4 BsAb cohorts, severe irAEs were infrequent. This observation is consistent with the design rationale of tumor-enriched dual-checkpoint engagement, but comparisons with historical nivolumab plus ipilimumab data should be interpreted cautiously because trial populations, regimens, AE definitions, and follow-up duration differ [8]. By leveraging cis-binding effects, these molecules are engineered to preferentially target tumor-infiltrating lymphocytes (TILs) that co-express both PD-1 and CTLA-4, thereby concentrating their activity within the tumor microenvironment and minimizing exposure to peripheral tissues that may express only a single target [36]. 

Our subgroup analysis suggested safety heterogeneity among different PD-1/CTLA-4 BsAb molecules, raising the possibility that individual agent-related factors may influence toxicity. Cadonilimab, for instance, demonstrated a relatively favorable safety profile, which may be related to its Fc-silent design. By eliminating FcgammaR-mediated antibody-dependent cell-mediated cytotoxicity (ADCC), this design could help prevent the non-specific depletion of peripheral T cells and preserve immune homeostasis [37, 38]. Conversely, molecules retaining a functional Fc domain, such as volrustomig (MEDI5752), were associated with a higher toxicity signal in our analysis, a finding consistent with early clinical reports [39]. Affinity tuning may represent another engineering strategy because reducing CTLA-4 affinity while maintaining PD-1 binding is designed to limit drug activity in normal tissues with low target density and promote preferential accumulation within the tumor microenvironment [40, 41]. Therefore, our findings support further investigation into how molecular engineering may influence the safety of dual PD-1/CTLA-4 blockade. However, these observations should be interpreted cautiously because cross-agent differences may also reflect differences in dose, trial phase, patient selection, treatment line, and concomitant therapy. Conclusions regarding severe TRAEs in the PD-1/CTLA-4 analysis should also be interpreted cautiously because Egger’s test suggested possible funnel plot asymmetry for this endpoint.

For PD-1/VEGF BsAbs, the currently available cohorts suggest numerically low pooled discontinuation rates in monotherapy settings, whereas higher rates of severe toxicity were observed in cohorts receiving chemotherapy-containing regimens. This pattern is biologically plausible because chemotherapy can independently contribute to hematologic, gastrointestinal, and systemic toxicities and may amplify toxicity when combined with VEGF pathway blockade [42, 43]. However, the observation cannot establish that chemotherapy was the sole driver of increased AE incidence, because chemotherapy-containing cohorts may differ from monotherapy cohorts in treatment line, disease biology, prior therapy exposure, baseline patient risk, and study design. Mechanistically, PD-1/VEGF BsAbs may still offer a rationale for tumor-microenvironment modulation through concurrent immune and angiogenic targeting [33, 44], but the present safety dataset cannot establish that this class is universally safer or that chemotherapy-free treatment is preferable for all patients. Similarly, chemotherapy-containing regimens should be interpreted separately from BsAb monotherapy whenever possible, because chemotherapy can independently increase hematologic toxicity, gastrointestinal toxicity, fatigue, and treatment discontinuation, thereby confounding attribution of AEs to the BsAb itself.

The irAE spectra provide additional descriptive insights. The irAEs associated with PD-1/CTLA-4 BsAbs predominantly involved cutaneous toxicities, endocrinopathies, and colitis, a pattern consistent with broader T-cell activation [45]. While our analysis suggests that severe irAEs were uncommon, clinicians must remain vigilant for delayed-onset events such as hypophysitis [15]. In contrast, PD-1/VEGF cohorts showed lower pooled overall irAE incidence in the available evidence, but this should not be interpreted as a direct comparative advantage because patient populations, study phases, and regimens differed. This observation also should not be interpreted as definitive evidence that VEGF inhibition directly reduces or does not amplify immunotoxicity. Vigilance for on-target VEGF-related toxicities, such as proteinuria and hypertension, remains essential, particularly in patients with cardiovascular comorbidities [46, 47]. 

These findings should not be used to select one BsAb class over another solely on the basis of pooled AE incidence. Instead, they highlight the importance of interpreting safety outcomes within the context of the specific agent, treatment regimen, patient population, study phase, and prior therapy exposure [48]. For PD-1/CTLA-4 BsAbs, future studies should clarify how molecular design, dose, and treatment setting influence immune-related toxicity. For PD-1/VEGF BsAbs, future trials should distinguish the safety profile of BsAb monotherapy from that of chemotherapy-containing combinations [15]. The inclusion of EGFR-mutated NSCLC cohorts deserves particular caution. Patients with EGFR-mutated disease are often enrolled after EGFR-TKI failure and may have distinct prior treatment exposure, disease biology, immune microenvironment, and baseline toxicity risks compared with unselected or driver-negative NSCLC populations. Therefore, AE rates from EGFR-mutated cohorts after EGFR-TKI failure should not be contrasted with those from first-line or immunotherapy-naive unselected populations as if the populations were exchangeable [49]. Future analyses should evaluate EGFR-mutated and driver-negative NSCLC separately when sufficient data become available.

The significant Egger’s test for grade 3 or higher TRAEs in the PD-1/CTLA-4 analysis deserves careful consideration. Although publication bias is one possible explanation for funnel plot asymmetry, several alternative explanations are also plausible in this context. First, small-study effects may be present because early-phase BsAb trials often have limited sample sizes, dose-escalation or dose-expansion designs, and more variable safety estimates. Second, substantial clinical heterogeneity may contribute to asymmetry, as the included PD-1/CTLA-4 cohorts differed in individual agents, molecular design, dose level, treatment line, concomitant therapy, and patient characteristics. Third, severe TRAE reporting may vary across early-phase and later-phase studies because of differences in AE ascertainment, attribution rules, and follow-up duration. Therefore, the significant Egger’s test should be interpreted as evidence of possible funnel plot asymmetry rather than definitive proof of publication bias [50]. This finding further supports cautious interpretation of the pooled grade 3 or higher TRAE incidence for PD-1/CTLA-4 BsAbs.

This study has several limitations that warrant consideration. First, substantial heterogeneity was observed in several key pooled analyses, which limits the robustness and clinical interpretability of some pooled incidence estimates. This heterogeneity likely reflects differences in study phase, sample size, follow-up duration, eligibility criteria, patient population, treatment line, molecular subtype, prior therapy exposure, treatment regimen, and individual BsAb agent. Although random-effects models and subgroup analyses were used to account for and explore heterogeneity, the limited number of studies within several subgroups precluded reliable meta-regression or definitive identification of heterogeneity sources. Therefore, pooled estimates, especially for all-grade TRAEs and overall irAEs, should be interpreted cautiously as descriptive summaries rather than precise incidence rates [51]. Second, no direct head-to-head randomized trials comparing PD-1/CTLA-4 with PD-1/VEGF BsAbs were available, and this study did not perform a formal between-class comparative meta-analysis. The two BsAb classes were synthesized through independent single-arm proportion meta-analyses. Therefore, cross-class differences in pooled estimates should be interpreted only as descriptive and hypothesis-generating observations, not as evidence of superiority, equivalence, or statistically significant between-class differences. Third, the included studies were clinically heterogeneous and included different treatment lines, molecular subgroups, EGFR-mutated post-TKI populations, treatment-naive populations, BsAb monotherapy cohorts, and chemotherapy-containing combination regimens. These factors are likely to influence the incidence and pattern of AEs independently of the BsAb class itself [52]. Fourth, the pooling of early-phase and late-phase studies may have contributed to heterogeneity and may limit the clinical interpretability of pooled safety estimates. Phase I/Ib and phase Ib/II studies are often designed to assess dose, tolerability, and preliminary activity; they may include dose-escalation or dose-expansion cohorts, broader dose ranges, smaller sample sizes, and more intensive AE monitoring. In contrast, phase II and phase III studies usually evaluate selected doses or regimens in more defined populations under more standardized protocols. Because the number of cohorts within phase-specific strata was limited, we were unable to perform reliable stratified meta-analyses by study phase for all endpoints. Therefore, the potential influence of study phase was addressed descriptively and should be considered when interpreting the pooled estimates. Fifth, potential publication bias or small-study effects should be considered. Although most publication-bias tests were non-significant, Egger’s test for grade 3 or higher TRAEs in the PD-1/CTLA-4 analysis was statistically significant (*p* = 0.024), indicating possible funnel plot asymmetry. This asymmetry may reflect publication bias, but it may also be attributable to small-study effects or substantial clinical heterogeneity among the included studies. Finally, a substantial portion of the current safety data originates from studies conducted predominantly in Asian populations, particularly for agents developed in China, and the generalizability of these findings to broader global populations remains to be confirmed.

Looking ahead, future research should prioritize the identification of predictive safety biomarkers. Investigating whether baseline levels of soluble VEGFR, the composition of the gut microbiome, or specific HLA genotypes can predict toxicities for PD-1/VEGF and PD-1/CTLA-4 BsAbs, respectively, would be a significant advance. Furthermore, as novel therapeutic classes emerge, exploring new combination strategies will be critical [53, 54]. The combination of BsAbs with antibody-drug conjugates (ADCs), for example, represents a key future direction. Navigating the challenge of enhancing efficacy while managing the potential for cumulative or overlapping toxicities from such novel pairings will be paramount.

## Conclusion

In conclusion, this systematic review and single-arm meta-analysis provides a descriptive synthesis of available safety data for PD-1/CTLA-4 and PD-1/VEGF BsAbs in NSCLC. Exploratory subgroup analyses suggested that AE incidence may vary according to individual BsAb agents and treatment regimens. However, the included evidence is clinically and methodologically heterogeneous, spanning early-phase and late-phase studies with different objectives, dose-selection strategies, patient populations, treatment lines, molecular subtypes, concomitant therapies, and AE reporting practices. Therefore, pooled incidence estimates should be interpreted cautiously as descriptive summaries rather than definitive safety rates applicable across all phases of clinical development or as direct comparative evidence between BsAb classes. The pooled estimates, particularly for PD-1/CTLA-4 grade 3 or higher TRAEs, should be interpreted cautiously because of potential small-study effects, publication bias, and clinical heterogeneity. Future prospective studies with larger homogeneous cohorts, standardized AE reporting, phase-specific analyses, and direct comparative designs are needed to clarify the relative contribution of agent-related and regimen-related factors to BsAb safety.

## Supplementary Information

Supplementary Material 1.

## Data Availability

All data generated or analysed during this study are included in this article. Further enquiries can be directed to the corresponding author.
