# Supplementary material for: Pooled safety profiles of bispecific antibodies targeting PD-1/CTLA-4 or PD-1/VEGF in non-small cell lung cancer: a systematic review and single-arm meta-analysis
Source: J Egypt Natl Canc Inst. 2026 Aug 5;38:54. doi: 10.1186/s43046-026-00387-2 (PMC13442781; doi:10.1186/s43046-026-00387-2)
Supplement: Supplementary file 1 [file 43046_2026_387_MOESM1_ESM.docx]

Table S1 Quality assessment of included studies by MINORS.

| Study | 1 | 2 | 3 | 4 | 5 | 6 | 7 | 8 | 9 | 10 | 11 | 12 | Total |
| --- | --- | --- | --- | --- | --- | --- | --- | --- | --- | --- | --- | --- | --- |
| Y. Zheng./2025 | 2 | 1 | 2 | 2 | 2 | 2 | 1 | 1 |  |  |  |  | 13 |
| Y. Zhang./2025 | 2 | 1 | 2 | 2 | 2 | 2 | 2 | 1 |  |  |  |  | 14 |
| L. Wang./2024 | 2 | 2 | 2 | 2 | 2 | 2 | 2 | 1 |  |  |  |  | 15 |
| Y. Zhao.a/2024 | 2 | 2 | 2 | 2 | 1 | 2 | 2 | 1 |  |  |  |  | 14 |
| D.R. Spigel./2024 | 2 | 1 | 2 | 2 | 1 | 2 | 2 | 1 |  |  |  |  | 13 |
| Y. Huang./2024 | 2 | 1 | 2 | 2 | 2 | 2 | 2 | 1 |  |  |  |  | 14 |
| Y. Zhao.b/2024 | 2 | 1 | 2 | 2 | 1 | 2 | 2 | 1 |  |  |  |  | 13 |
| A. Xiong./2023 | 2 | 2 | 2 | 2 | 2 | 2 | 2 | 1 |  |  |  |  | 15 |
| B. Chen./2023 | 2 | 1 | 2 | 2 | 1 | 2 | 2 | 1 |  |  |  |  | 13 |
| Y. Zhao.a/2023 | 2 | 1 | 2 | 2 | 1 | 2 | 2 | 1 |  |  |  |  | 13 |
| L. Wang./2024 | 2 | 1 | 2 | 2 | 1 | 2 | 2 | 1 |  |  |  |  | 13 |
| Y. Wu./2024 | 2 | 2 | 2 | 2 | 1 | 2 | 2 | 1 |  |  |  |  | 14 |
| Y. Zhao.b/2023 | 2 | 2 | 2 | 2 | 1 | 2 | 2 | 1 |  |  |  |  | 14 |

1.A stated aim of the study; 2.Inclusion of consecutive patients; 3.Prospective collection of data; 4.End point appropriate to the study aim; 5.Unbiased evaluation of endpoints; 6.Follow-up period appropriate to the major endpoint; 7.Loss to follow up not exceeding 5%; 8.Prospective calculation of the sample size; 9.A control group having the gold standard intervention; 10.Contemporary groups; 11.Baseline equivalence of groups; 12.Statistical analyses adapted to the study design.

Abbreviations: Methodological index for non-randomized studies (MINORS)

Table S2 P values of Begg’s and Egger’s tests for TRAE.

|  | PD-1/CTLA-4 TRAE | PD-1/VEGF TRAE | PD-1/CTLA-4≥3grade TRAE | PD-1/VEGF≥3grade TRAE | PD-1/CTLA-4 Serious TRAE | PD-1/VEGF Serious TRAE | PD-1/CTLA-4 discontinuation TRAE | PD-1/VEGF discontinuation TRAE |
| --- | --- | --- | --- | --- | --- | --- | --- | --- |
| Begg’s test | 0.228 | 0.764 | 0.101 | 0.548 | 0.213 | 0.462 | 0.536 | 0.764 |
| Egger’s test | 0.664 | 0.417 | 0.024 | 0.330 | 0.127 | 0.443 | 0.440 | 0.886 |

Abbreviations: programmed cell death-1 (PD-1); cytotoxic t-lymphocyte–associated protein 4 (CTLA-4); treatment-related adverse events (TRAE).

Table S3 P values of Begg’s and Egger’s tests for irAE.

|  | PD-1/CTLA-4 irAE | PD-1/VEGF irAE | PD-1/CTLA-4≥3grade irAE | PD-1/VEGF≥3grade irAE |
| --- | --- | --- | --- | --- |
| Begg’s test | 0.640 | 0.230 | 0.858 | 0.038 |
| Egger’s test | 0.185 | 0.168 | 0.260 | 0.110 |

Abbreviations: programmed cell death-1 (PD-1); cytotoxic t-lymphocyte–associated protein 4 (CTLA-4); immune-related adverse events (irAE).

**A.****B.**

**Figure S1 Quality assessment of the included studies. (A) The details of bias assessment. (B) The summary of bias assessment.**

**A.****B.**

**C.****D.**

**Figure S2 Funnel plot of PD-1/CTLA-4–related TRAEs. (A) Overall TRAE. (B)** ≥3grade TRAE. (C) Serious TRAE. **(D)** Discontinuation TRAE.

**A.****B.**

**C.****D.**

**Figure S3 Funnel plot of** PD-1/VEGF**–related TRAEs. (A) Overall TRAE. (B)** ≥3grade TRAE. (C) Serious TRAE. **(D)** Discontinuation TRAE.

**A.****B.**

**C.****D.**

**Figure S4 Funnel plot of irAEs. (A) PD-1/****CTLA-4 Overall irAE. (B) PD-1/CTLA-4** ≥3grade **ir**AE. (C) PD-1/VEGF **Overall irAE**. **(D)** PD-1/VEGF ≥3grade **ir**AE.

**A.****B.**

**C.****D.**

**Figure S5 Sensitivity analysis plots for PD-1/ CTLA-4–related TRAEs. (A) Overall TRAE. (B)** ≥3grade TRAE. (C) Serious TRAE. **(D)** Discontinuation TRAE.

**A.****B.**

**C.****D.**

**Figure S6 Sensitivity analysis plots for** PD-1/VEGF**–related TRAEs. (A) Overall TRAE. (B)** ≥3grade TRAE. (C) Serious TRAE. **(D)** Discontinuation TRAE.

**A.****B.**

**C.****D.**

**Figure S7 Sensitivity Analysis Plots for irAEs. (A) PD-1/CTLA-4 Overall irAE. (B) PD-1/CTLA-4** ≥3grade **ir**AE. (C) PD-1/VEGF **Overall irAE**. **(D)** PD-1/VEGF ≥3grade **ir**AE.

**A.****B.**

**C.****D.**

**Figure S8. Forest plot of subgroup analyses of TRAEs across different PD-1/CTLA-4 inhibitors. (A) Overall TRAE. (B)** ≥3grade TRAE. (C) Serious TRAE. **(D)** Discontinuation TRAE.

**A.****B.**

**C.**

**Figure S9. Forest plot of subgroup analyses of TRAEs across different** PD-1/VEGF **inhibitors. (A) Overall TRAE. (B)** ≥3grade TRAE. (C) Discontinuation TRAE.

**A.****B.**

**C.****D.**

**Figure S10. Forest plot of subgroup analyses of irAEs by individual agents. (A) PD-1/CTLA-4 Overall irAE. (B) PD-1/CTLA-4** ≥3grade **ir**AE. (C) PD-1/VEGF **Overall irAE**. **(D)** PD-1/VEGF ≥3grade **ir**AE.

**A.****B.**

**C.****D.**

**Figure S11. Forest plot of subgroup analyses of PD-1/CTLA-4–related TRAEs by lines of therapy. (A) Overall TRAE. (B)** ≥3grade TRAE. (C) Serious TRAE. **(D)** Discontinuation TRAE.

**A.****B.**

**C.****D.**

**Figure S12. Forest plot of subgroup analyses of PD-1/** VEGF**–related TRAEs by lines of therapy. (A) Overall TRAE. (B)** ≥3grade TRAE. (C) Serious TRAE. **(D)** Discontinuation TRAE.

**A.****B.**

**C.****D.**

**Figure S13. Forest plot of subgroup analyses of irAEs by lines of therapy. (A) PD-1/CTLA-4 Overall irAE. (B) PD-1/CTLA-4** ≥3grade **ir**AE. (C) PD-1/VEGF **Overall irAE**. **(D)** PD-1/VEGF ≥3grade **ir**AE.

**A.****B.**

**C.****D.**

**Figure S14. Forest plot of subgroup analyses of PD-1/CTLA-4–related TRAEs by treatment regimens. (A) Overall TRAE. (B)** ≥3grade TRAE. (C) Serious TRAE. **(D)** Discontinuation TRAE.

**A.****B.**

**C.****D.**

**Figure S15. Forest plot of subgroup analyses of PD-1/** VEGF**–related TRAEs by treatment regimens. (A) Overall TRAE. (B)** ≥3grade TRAE. (C) Serious TRAE. **(D)** Discontinuation TRAE.

**A.****B.**

**C.****D.**

**Figure S16. Forest plot of subgroup analyses of irAEs by treatment regimens. (A) PD-1/CTLA-4 Overall irAE. (B) PD-1/CTLA-4** ≥3grade **ir**AE. (C) PD-1/VEGF **Overall irAE**. **(D)** PD-1/VEGF ≥3grade **ir**AE.
